# Supplementary material for: Intensity and Duration of Vibration Emissions during Shipping as Interacting Factors on the Quality of Boar Semen Extended in Beltsville Thawing Solution
Source: Animals (Basel). 2023 Mar 6;13(5):952. doi: 10.3390/ani13050952 (PMC10000154; doi:10.3390/ani13050952)
Supplement: Supplementary file 1 [file animals-13-00952-s001.zip › animals-2239439-supplementary.pdf]

Table S1. Summary of total sperm motility data collected on day 1 after treatment.

	$D_i = 0.5$		$D_i = 1.0$		$D_i = 1.5$		$D_i = 2.0$		$D_i = 3.0$		$D_i = 4.0$		$D_i = 5.0$		$D_i = 6.0$	
**Duration**	**TSM**	**Δ-TSM**	**TSM**	**Δ-TSM**	**TSM**	**Δ-TSM**	**TSM**	**Δ-TSM**	**TSM**	**Δ-TSM**	**TSM**	**Δ-TSM**	**TSM**	**Δ-TSM**	**TSM**	**Δ-TSM**
0.0	77.3 ± 4.1	0	84.3 ± 4.4	0	82.6 ± 3.8	0	80 ± 7.4	0	81.7 ± 3.8	0	78.9 ± 10.3	0	77.2 ± 5.2	0	80.6 ± 4.7	0
0.5	75.3 ± 7.7	-2 ± 2.1	82.8 ± 6.4	-1.5 ± 1.8	79.2 ± 6.5	-3.4 ± 1.3	77.5 ± 6.2	-2.5 ± 1	82.8 ± 3.7	1.1 ± 1.7	78.5 ± 8.2	-0.4 ± 1.1	77.7 ± 4.9	0.5 ± 0.4	78 ± 5.7	-2.6 ± 1.9
1.0	76.5 ± 5.3	-0.8 ± 0.8	82.6 ± 4.4	-1.7 ± 0.3	79.8 ± 6.1	-2.8 ± 1.3	72.8 ± 10.4	-7.2 ± 2.3	79.3 ± 6.9	-2.4 ± 2	76.9 ± 9.7	-2 ± 0.7	77.1 ± 3.4	-0.1 ± 1.8	77.4 ± 2.6	-3.2 ± 1.8
2.0	75.1 ± 5.1	-2.2 ± 0.8	79.5 ± 8	-4.7 ± 2.1	79.9 ± 6.5	-2.7 ± 1.4	73.5 ± 6.9	-6.4 ± 1.6	74.6 ± 7.6	-7.1 ± 2.6	75.6 ± 9.7	-3.3 ± 1.4	74.3 ± 2.7	-2.9 ± 1.4	78.3 ± 3.1	-2.3 ± 2.4
3.0	75.5 ± 3.3	-1.8 ± 0.6	80.4 ± 5.1	-3.9 ± 1.5	78.3 ± 7.8	-4.4 ± 2.3	73.4 ± 8.1	-6.6 ± 2.4	78.7 ± 5.4	-3 ± 2	73.4 ± 9.4	-5.5 ± 1.6	76.7 ± 3.8	-0.4 ± 2.7	75.5 ± 5.2	-5.1 ± 1.3
4.0	74.5 ± 5.3	-2.8 ± 0.7	81.5 ± 3.7	-2.8 ± 0.7	80.4 ± 6.9	-2.3 ± 1.4	71.9 ± 6.3	-8 ± 1.4	78.2 ± 6.8	-3.5 ± 2.6	74.3 ± 8.4	-4.6 ± 1.2	73.3 ± 3.6	-3.9 ± 2.4	73.9 ± 5	-6.7 ± 1.4
5.0	74.3 ± 4.7	-2.9 ± 0.3	79.4 ± 4	-4.8 ± 2.7	74 ± 11	-8.6 ± 3.2	68.2 ± 5.9	-11.8 ± 1.9	76.8 ± 7.1	-4.9 ± 2.6	74.4 ± 7.9	-4.6 ± 1.7	74.4 ± 4.5	-2.8 ± 0.8	71.6 ± 8.2	-9 ± 3.3
6.0	75.5 ± 3.5	-1.8 ± 0.4	74.3 ± 5.9	-10 ± 1.2	74 ± 9.4	-8.7 ± 2.6	69.1 ± 3.3	-10.9 ± 3.4	75.9 ± 5.3	-5.8 ± 1.4	71.3 ± 10.6	-7.6 ± 1.5	74 ± 4.5	-3.2 ± 2.4	73.7 ± 2.9	-6.8 ± 2.4
7.0	77.1 ± 3.2	-0.2 ± 1.9	75.6 ± 8.2	-8.7 ± 2.4	74.9 ± 9.7	-7.7 ± 2.7	68.9 ± 11.9	-11.1 ± 2.1	75.4 ± 4.6	-6.2 ± 1.4	73.6 ± 8.2	-5.3 ± 1	72.3 ± 4.8	-4.8 ± 2.2	73.6 ± 7.2	-7 ± 2.6
8.0	77.9 ± 6.4	0.6 ± 1.5	79.5 ± 4.7	-4.8 ± 1	75.4 ± 7.7	-7.2 ± 1.9	67.9 ± 9.8	-12.1 ± 3.9	73.3 ± 5.4	-8.4 ± 2	71.9 ± 9.5	-7 ± 1.9	73.3 ± 6.8	-3.9 ± 2.2	70.4 ± 4.6	-10.2 ± 1.1
9.0	75.8 ± 4.2	-1.5 ± 2.1	77.9 ± 4.3	-6.3 ± 0.3	75.6 ± 8.2	-7 ± 2.4	71.8 ± 5	-8.2 ± 1.4	74.7 ± 4.5	-7 ± 1.2	68.2 ± 13.5	-10.7 ± 4.9	72.4 ± 6	-4.8 ± 0.6	73.1 ± 6.8	-7.5 ± 1.9
10.0	79.2 ± 4.3	1.9 ± 1.3	77.7 ± 5.3	-6.5 ± 1.8	73.3 ± 7.5	-9.3 ± 2.4	70.7 ± 5.9	-9.3 ± 2.1	70.9 ± 8.4	-10.7 ± 2.9	67 ± 6.8	-11.9 ± 2.5	69.2 ± 3.9	-8 ± 3.7	65.5 ± 3.6	-15.1 ± 1.5
11.0	76.1 ± 5.3	-1.2 ± 0.9	77.6 ± 5	-6.7 ± 0.7	75.3 ± 7.6	-7.3 ± 1.8	67.7 ± 7.5	-12.2 ± 5	63.8 ± 9.6	-17.9 ± 3.1	66.8 ± 9.4	-12.1 ± 1.7	68.5 ± 7.4	-8.7 ± 3.4	66.3 ± 7	-14.2 ± 2.3
12.0	74 ± 3.3	-3.2 ± 0.9	72.3 ± 5.2	-12 ± 0.5	74.5 ± 8	-8.1 ± 2.2	67.6 ± 6.3	-12.4 ± 1.7	69.3 ± 7.9	-12.4 ± 2.1	66.4 ± 12.8	-12.5 ± 2.7	62 ± 3.5	-15.2 ± 2.2	67.7 ± 11.7	-12.9 ± 3.8

TSM – total sperm motility (%, means ± SD), Δ-TSM - difference to unshaken control group (%, means ± SE), Duration (h).

Table S2. Summary of total sperm motility data collected on day 2 after treatment.

	$D_i = 0.5$		$D_i = 1.0$		$D_i = 1.5$		$D_i = 2.0$		$D_i = 3.0$		$D_i = 4.0$		$D_i = 5.0$		$D_i = 6.0$	
**Duration**	**TSM**	**Δ-TSM**	**TSM**	**Δ-TSM**	**TSM**	**Δ-TSM**	**TSM**	**Δ-TSM**	**TSM**	**Δ-TSM**	**TSM**	**Δ-TSM**	**TSM**	**Δ-TSM**	**TSM**	**Δ-TSM**
0.0	79.6 ± 6.1	0	79.8 ± 3.5	0	78.5 ± 6.1	0	77.1 ± 8.1	0	81.4 ± 5.8	0	76.2 ± 8.9	0	73.7 ± 2.3	0	78.6 ± 5.8	0
0.5	78.3 ± 4.5	-1.3 ± 1.2	83.9 ± 4.5	4.1 ± 2.2	80.5 ± 7	2 ± 1.1	75.5 ± 9.9	-1.6 ± 2.1	82.6 ± 4.3	1.2 ± 1.1	76.8 ± 10.1	0.6 ± 1	75 ± 4.1	1.3 ± 2.3	76.9 ± 5	-1.7 ± 1
1.0	79.8 ± 5.3	0.2 ± 0.8	81.4 ± 2.9	1.6 ± 0.5	77.6 ± 7.7	-0.9 ± 1.7	74.2 ± 6.9	-2.9 ± 2.6	80.6 ± 6.3	-0.8 ± 1.9	77.7 ± 8.1	1.5 ± 1.1	79.1 ± 4.9	5.5 ± 3.9	77.4 ± 3.3	-1.2 ± 2.8
2.0	76.7 ± 5.7	-2.9 ± 0.6	81.8 ± 4.8	2 ± 2.8	81.3 ± 5	2.8 ± 1.2	75.9 ± 7.3	-1.2 ± 1.2	79.9 ± 5.4	-1.5 ± 2.7	75.9 ± 8.4	-0.4 ± 1	70 ± 2.4	-3.7 ± 1.9	76.1 ± 5.5	-2.5 ± 1.1
3.0	75.5 ± 6.3	-4.1 ± 0.6	82.9 ± 3.2	3.1 ± 0.6	78.5 ± 7.1	0 ± 1.5	75.6 ± 4.2	-1.5 ± 2.2	77.9 ± 7.1	-3.5 ± 2.5	74.4 ± 8.2	-1.9 ± 0.6	70.9 ± 2.6	-2.8 ± 1.4	73.8 ± 8.3	-4.8 ± 1.3
4.0	75.4 ± 10	-4.2 ± 2.9	81.7 ± 4.5	1.9 ± 2.6	78.8 ± 4.4	0.4 ± 1.4	73 ± 4.1	-4.1 ± 2.5	76 ± 6	-5.4 ± 2.1	74 ± 9.1	-2.3 ± 0.8	70.4 ± 5.1	-3.3 ± 3.6	71.2 ± 7.3	-7.4 ± 2.7
5.0	75.2 ± 5.3	-4.4 ± 0.8	80.7 ± 6.3	0.9 ± 2.7	75.6 ± 10.1	-2.9 ± 1.9	70.5 ± 4.1	-6.6 ± 1.9	78.2 ± 5.5	-3.2 ± 1.2	73.3 ± 8.8	-2.9 ± 1.4	73.8 ± 4.5	0.1 ± 3.3	71.6 ± 7.5	-7.1 ± 1.7
6.0	75.7 ± 6.2	-4 ± 2	76.4 ± 5.1	-3.4 ± 2.6	75.4 ± 8.2	-3.1 ± 2.4	69.6 ± 6	-7.4 ± 2.4	79.5 ± 4	-1.9 ± 1.2	71.2 ± 9.1	-5.1 ± 1.2	71.9 ± 9.4	-1.7 ± 6	69.2 ± 3	-9.4 ± 1.6
7.0	76.9 ± 1.8	-2.8 ± 2.9	80.7 ± 3.9	0.9 ± 2.3	75.9 ± 9.9	-2.6 ± 1.9	73.8 ± 8.5	-3.2 ± 2.8	74.6 ± 5.6	-6.8 ± 1.7	73.8 ± 7.4	-2.4 ± 1	72.8 ± 4.6	-0.9 ± 3.9	69.7 ± 5.7	-8.9 ± 1.4
8.0	75.9 ± 5.9	-3.7 ± 2	79.2 ± 6.8	-0.6 ± 2.6	74.4 ± 7.9	-4 ± 1.3	72 ± 4.5	-5 ± 3.2	72.4 ± 7.3	-9 ± 1.9	70.3 ± 8.1	-6 ± 1.1	69.6 ± 7	-4 ± 4.1	69.8 ± 3.1	-8.8 ± 2.9
9.0	74.8 ± 8.1	-4.8 ± 2.8	79.4 ± 3.9	-0.4 ± 1.7	73.8 ± 9.4	-4.7 ± 2.2	74.4 ± 8.9	-2.7 ± 2.3	72.6 ± 5.3	-8.8 ± 1.5	71.6 ± 8.7	-4.7 ± 1.1	71.5 ± 6.7	-2.2 ± 5.1	67.9 ± 5.4	-10.7 ± 1.3
10.0	73.2 ± 3.7	-6.5 ± 1.5	77.8 ± 6.8	-2 ± 3.9	71.3 ± 7.1	-7.2 ± 2.4	70.5 ± 6	-6.6 ± 2.7	72 ± 6.7	-9.4 ± 1.7	68.9 ± 7.5	-7.3 ± 1.7	70.6 ± 6.4	-3.1 ± 4.9	65.4 ± 8.6	-13.2 ± 1.6
11.0	75.1 ± 7.3	-4.5 ± 2.6	78.5 ± 6.2	-1.3 ± 1.6	74.1 ± 8.1	-4.4 ± 1.7	68.7 ± 8.4	-8.4 ± 4.4	64.6 ± 5.5	-16.8 ± 2.6	70.8 ± 9.5	-5.4 ± 1.5	64.3 ± 7.7	-9.3 ± 3.6	63.1 ± 11.7	-15.5 ± 3.2
12.0	76 ± 7.1	-3.7 ± 0.6	75.3 ± 8.6	-4.5 ± 3.5	74.6 ± 7.9	-3.9 ± 2.1	64.9 ± 4.4	-12.2 ± 4.3	66.9 ± 6.8	-14.5 ± 4.1	64.9 ± 9.5	-11.3 ± 1.6	66.9 ± 6.1	-6.8 ± 2.2	62.6 ± 14.9	-16 ± 4.2

TSM – total sperm motility (%, means ± SD), Δ-TSM - difference to unshaken control group (%, means ± SE), Duration (h).

Table S3. Summary of total sperm motility data collected on day 3 after treatment.

	D_i = 0.5		D_i = 1.0		D_i = 1.5		D_i = 2.0		D_i = 3.0		D_i = 4.0		D_i = 5.0		D_i = 6.0	
**Duration**	**TSM**	**Δ-TSM**	**TSM**	**Δ-TSM**	**TSM**	**Δ-TSM**	**TSM**	**Δ-TSM**	**TSM**	**Δ-TSM**	**TSM**	**Δ-TSM**	**TSM**	**Δ-TSM**	**TSM**	**Δ-TSM**
0.0	79.3 ± 6.1	0	79 ± 4.8	0	79.2 ± 7.6	0	77.3 ± 9.2	0	83.4 ± 2.8	0	75 ± 9.2	0	75.8 ± 6.1	0	79.6 ± 5.6	0
0.5	79.6 ± 6.5	0.4 ± 1.4	78.1 ± 3.5	-0.9 ± 3.3	78.7 ± 7	-0.5 ± 1.3	76.2 ± 6.2	-1 ± 1.4	78.3 ± 6.8	-5.2 ± 2	74.5 ± 10.7	-0.4 ± 1.6	78.9 ± 6.8	3.1 ± 0.6	77.3 ± 5.2	-2.3 ± 1.5
1.0	79.5 ± 2	0.3 ± 2.6	79.7 ± 6.1	0.7 ± 2	78 ± 9.1	-1.2 ± 1.1	76.6 ± 7.3	-0.7 ± 1.7	78 ± 4.5	-5.5 ± 1.1	74.8 ± 9.1	-0.2 ± 0.8	77 ± 4.4	1.2 ± 1	75.2 ± 7.5	-4.4 ± 1.8
2.0	76.3 ± 8.5	-3 ± 2.2	76.7 ± 4.6	-2.3 ± 3.7	78.4 ± 6.1	-0.8 ± 1.1	75.5 ± 6.9	-1.8 ± 1.9	77.8 ± 6	-5.6 ± 1.7	72.8 ± 9.3	-2.2 ± 1.7	77.9 ± 6.6	2.1 ± 4.5	73.4 ± 10.9	-6.2 ± 3.8
3.0	76.8 ± 7.5	-2.5 ± 0.9	79.8 ± 6.2	0.9 ± 1.7	75.4 ± 9.4	-3.8 ± 1.2	73.6 ± 5.8	-3.7 ± 1.8	75.9 ± 3.8	-7.5 ± 0.7	72.9 ± 10.6	-2.1 ± 1.2	75.9 ± 3.2	0.1 ± 2.8	69.5 ± 7.5	-10.1 ± 2.8
4.0	75.9 ± 5.9	-3.3 ± 0.6	83.9 ± 2.6	4.9 ± 1.6	75.2 ± 10	-4 ± 1.5	71.2 ± 5.7	-6 ± 1.7	75.4 ± 5.5	-8 ± 1.8	70.8 ± 11.7	-4.2 ± 1.7	69.1 ± 5.2	-6.6 ± 4.4	69.2 ± 13.1	-10.4 ± 4.2
5.0	76.5 ± 7	-2.8 ± 1.8	79.8 ± 4	0.8 ± 0.5	73.1 ± 12.7	-6.1 ± 3.5	71.6 ± 4.4	-5.7 ± 2.4	77.7 ± 6	-5.7 ± 1.9	74.3 ± 11.6	-0.7 ± 2.2	73.2 ± 3.5	-2.5 ± 2.9	71.1 ± 9.5	-8.6 ± 3.5
6.0	75.8 ± 6.3	-3.4 ± 2	77.4 ± 7.3	-1.6 ± 1.7	73.2 ± 9.6	-6 ± 1.7	71 ± 7.7	-6.2 ± 2.4	75.6 ± 7.1	-7.8 ± 2.2	69.1 ± 12.3	-5.9 ± 2.5	68.6 ± 6.4	-7.1 ± 2.2	69.6 ± 5.8	-10 ± 2.8
7.0	73.9 ± 6	-5.4 ± 0.1	78.8 ± 3.6	-0.1 ± 2.2	75.1 ± 9.4	-4.1 ± 2.5	70.2 ± 9.6	-7.1 ± 2.7	74.8 ± 6	-8.6 ± 2.2	69.9 ± 9.9	-5.1 ± 1.8	76.2 ± 6.2	0.5 ± 1	64.1 ± 9.7	-15.5 ± 3.4
8.0	69.8 ± 3.9	-9.5 ± 3.5	76.2 ± 5.8	-2.8 ± 3.6	75.9 ± 10.1	-3.3 ± 1.3	69.2 ± 7.6	-8.1 ± 2.3	73 ± 7.1	-10.4 ± 2.2	69.2 ± 11.1	-5.8 ± 2	71.8 ± 5.7	-3.9 ± 0.2	67.7 ± 5.3	-11.9 ± 2.2
9.0	76.7 ± 7.5	-2.6 ± 1.1	76.7 ± 5.4	-2.3 ± 3.1	73.7 ± 9.2	-5.5 ± 1	71.3 ± 7.1	-5.9 ± 2.3	75.1 ± 2.8	-8.3 ± 0.9	68.9 ± 9.5	-6.1 ± 1.9	73.2 ± 5.5	-2.5 ± 1.7	65 ± 10.8	-14.6 ± 3.9
10.0	75.1 ± 2.6	-4.2 ± 3.7	76.3 ± 8.6	-2.7 ± 2.4	72.9 ± 5.9	-6.3 ± 1.5	71.7 ± 7.2	-5.5 ± 2.2	72.7 ± 7.7	-10.7 ± 2.4	67.6 ± 10.6	-7.4 ± 2.1	67.2 ± 4.9	-8.5 ± 1.4	61.1 ± 12.4	-18.5 ± 4.9
11.0	73.1 ± 5.3	-6.1 ± 1.4	76.4 ± 5.4	-2.6 ± 3	73.6 ± 9.3	-5.6 ± 1	66.7 ± 7.2	-10.6 ± 3.3	59.9 ± 9.1	-23.6 ± 3.8	63.1 ± 10.9	-11.9 ± 2.7	64.6 ± 7.1	-11.1 ± 4.2	61.8 ± 15.8	-17.9 ± 5.4
12.0	73.3 ± 3.8	-5.9 ± 1.5	68.6 ± 4.9	-10.4 ± 1.4	72.7 ± 11	-6.5 ± 2.3	65.1 ± 6.1	-12.1 ± 2.5	68.4 ± 9	-15 ± 3.4	62.2 ± 12	-12.8 ± 2.4	62.3 ± 4.9	-13.4 ± 5	60.7 ± 15.6	-19 ± 5.1

TSM – total sperm motility (%, means ± SD), Δ-TSM - difference to unshaken control group (%, means ± SE), Duration (h).

Table S4. Summary of total sperm motility data collected on day 4 after treatment.

	D_i = 0.5		D_i = 1.0		D_i = 1.5		D_i = 2.0		D_i = 3.0		D_i = 4.0		D_i = 5.0		D_i = 6.0	
**Duration**	**TSM**	**Δ-TSM**	**TSM**	**Δ-TSM**	**TSM**	**Δ-TSM**	**TSM**	**Δ-TSM**	**TSM**	**Δ-TSM**	**TSM**	**Δ-TSM**	**TSM**	**Δ-TSM**	**TSM**	**Δ-TSM**
0.0	75.5 ± 3.7	0	80.7 ± 6.5	0	78.4 ± 6.9	0	74.2 ± 9.4	0	79.4 ± 4.2	0	75.9 ± 7.8	0	79.3 ± 5.8	0	77.1 ± 6.9	0
0.5	75.8 ± 3.2	0.4 ± 0.3	81.1 ± 5	0.4 ± 0.9	78.8 ± 9.3	0.4 ± 1.8	74.6 ± 8.9	0.5 ± 1.8	79.9 ± 6.4	0.4 ± 1.9	75.8 ± 8.8	-0.1 ± 1.2	74.4 ± 5	-4.9 ± 1.3	75 ± 8.4	-2.1 ± 1.2
1.0	73.9 ± 4.2	-1.5 ± 0.3	81.2 ± 6.8	0.5 ± 0.2	77.3 ± 7.2	-1.1 ± 2.2	73.4 ± 6.9	-0.8 ± 1.6	78.4 ± 7.9	-1 ± 2.1	77.1 ± 7.2	1.2 ± 1.3	76.1 ± 6.9	-3.2 ± 0.6	75.7 ± 6.7	-1.3 ± 2.3
2.0	74.8 ± 5.3	-0.7 ± 1.4	78.9 ± 4.2	-1.8 ± 1.4	75 ± 6.5	-3.4 ± 2.2	74.5 ± 5.4	0.4 ± 2	76.4 ± 6	-3 ± 1.4	72.7 ± 8.2	-3.2 ± 0.8	72.9 ± 5.9	-6.4 ± 1.6	73.5 ± 8.4	-3.6 ± 1.6
3.0	78 ± 7.6	2.5 ± 2.5	82.2 ± 6	1.5 ± 1.6	78.1 ± 7.2	-0.3 ± 0.8	72.5 ± 6.1	-1.7 ± 2.2	74 ± 8.8	-5.4 ± 2.2	72.9 ± 6.9	-3 ± 0.9	78 ± 9.4	-1.3 ± 2.1	70.9 ± 10.4	-6.2 ± 3
4.0	72.9 ± 4.1	-2.5 ± 0.5	79.7 ± 6.8	-1 ± 2.4	75.1 ± 8.1	-3.3 ± 2.8	68.4 ± 6	-5.7 ± 3	72.6 ± 8.9	-6.8 ± 3.4	73.3 ± 7.8	-2.6 ± 0.7	71.8 ± 5.6	-7.4 ± 0.2	70.4 ± 10.8	-6.7 ± 2.7
5.0	76.4 ± 7.5	1 ± 2.3	79 ± 5.3	-1.7 ± 1.2	77 ± 7	-1.4 ± 1.2	71.2 ± 4.8	-3 ± 2.3	74.4 ± 6.9	-5 ± 2.2	73.6 ± 7.1	-2.3 ± 0.5	73 ± 7.6	-6.3 ± 1.9	71.3 ± 12.5	-5.8 ± 2.5
6.0	75.9 ± 4.4	0.4 ± 1.3	75.3 ± 6.9	-5.4 ± 0.7	75 ± 11.3	-3.4 ± 2.7	69.2 ± 8.8	-5 ± 1.9	75.2 ± 5.2	-4.2 ± 1.5	71.9 ± 9.5	-3.9 ± 1.5	72.4 ± 5	-6.9 ± 2.4	66.1 ± 8	-10.9 ± 2.9
7.0	75 ± 4	-0.5 ± 0.2	78 ± 4.2	-2.7 ± 1.4	76.3 ± 8.1	-2.1 ± 1	71.1 ± 9.9	-3 ± 2.7	74.6 ± 7.7	-4.8 ± 2.1	71 ± 8	-4.8 ± 1.7	74.6 ± 2.1	-4.7 ± 2.3	63.6 ± 14.7	-13.5 ± 4.3
8.0	70.8 ± 6.7	-4.6 ± 4.5	79.1 ± 6.7	-1.6 ± 0.3	74.1 ± 7.4	-4.3 ± 1.7	67.2 ± 7.8	-6.9 ± 2.8	69.6 ± 9	-9.8 ± 2.7	71.7 ± 9.3	-4.2 ± 1.3	66.7 ± 8.3	-12.6 ± 1.9	65.5 ± 11.4	-11.6 ± 2.7
9.0	77.5 ± 6.6	2.1 ± 1.8	79 ± 5.1	-1.7 ± 1.1	72.1 ± 10	-6.3 ± 2.4	67.9 ± 8.2	-6.2 ± 1.8	71.5 ± 4.7	-7.9 ± 1.3	71.9 ± 9.7	-4 ± 1.6	78.1 ± 3	-1.1 ± 2.3	64.3 ± 16.8	-12.7 ± 5.5
10.0	77.1 ± 6.3	1.6 ± 2.1	77.8 ± 8.9	-2.9 ± 2.1	72.4 ± 7.8	-6.1 ± 1.8	68.3 ± 9	-5.9 ± 1.8	73 ± 6.2	-6.4 ± 1.7	69 ± 7.8	-6.9 ± 0.5	69.2 ± 9.1	-10.1 ± 3.5	60 ± 20.6	-17.1 ± 6.9
11.0	76.6 ± 5	1.1 ± 1.1	77.7 ± 8.5	-3 ± 3.1	72.2 ± 9.1	-6.2 ± 2.3	66.4 ± 7.8	-7.7 ± 3.8	62.8 ± 8.9	-16.7 ± 2.6	68.5 ± 7	-7.4 ± 1.6	66.7 ± 2.6	-12.6 ± 4.1	55.1 ± 19.1	-21.9 ± 5.8
12.0	75.4 ± 2.9	0 ± 0.7	72.9 ± 7.7	-7.8 ± 1	74.6 ± 10.1	-3.8 ± 2.7	63.9 ± 7.2	-10.2 ± 2.8	67.7 ± 5.2	-11.7 ± 2.5	64.3 ± 9.9	-11.6 ± 1.9	62.8 ± 7.4	-16.4 ± 7.1	56.8 ± 25.4	-20.3 ± 8.9

TSM – total sperm motility (%, means ± SD), Δ-TSM - difference to unshaken control group (%, means ± SE), Duration (h).
